# A Dataset for Forestry Pest Identification

**DOI:** 10.3389/fpls.2022.857104

**Published:** 2022-07-14

**Authors:** Bing Liu, Luyang Liu, Ran Zhuo, Weidong Chen, Rui Duan, Guishen Wang

**Affiliations:** ^1^School of Computer Science and Engineering, Changchun University of Technology, Changchun, China; ^2^College of Computer Science and Technology, Jilin University, Changchun, China

**Keywords:** forestry pest identification, deep learning, forestry pest dataset, object detection, transformer

## Abstract

The identification of forest pests is of great significance to the prevention and control of the forest pests' scale. However, existing datasets mainly focus on common objects, which limits the application of deep learning techniques in specific fields (such as agriculture). In this paper, we collected images of forestry pests and constructed a dataset for forestry pest identification, called Forestry Pest Dataset. The Forestry Pest Dataset contains 31 categories of pests and their different forms. We conduct several mainstream object detection experiments on this dataset. The experimental results show that the dataset achieves good performance on various models. We hope that our Forestry Pest Dataset will help researchers in the field of pest control and pest detection in the future.

## 1. Introduction

It is well known that the untimely control of pests will cause serious damage and loss of commercial crops (Estruch et al., 1997). In recent years, the scope and extent of forestry pest events in China have increased dramatically, resulting in huge economic losses (Gandhi et al., 2018; FAO, 2020). The identification and detection of pests play a crucial role in agricultural pest control, providing a strong guarantee for crop yield growth and the agricultural economy (Fina et al., 2013). Traditional forestry pest identification relies on a small number of forestry protection workers and insect researchers (Al-Hiary et al., 2011), generally based on the appearance of insects, through manual inspection, visual inspection of insect wings, antennae, mouthparts, feet, etc. to complete the identification of insects, but Due to the wide variety of pests and the small differences between the species, this method has major defects in practice. With the development of machine learning and computer vision technology, automatic pest identification has received more and more attention.

Most of the early pest identification work was done by using a machine learning framework, which consists of two modules: (1) hand-made feature extractors based on GIST (Torralba et al., 2003), Scale-Invariant Feature Transform(SIFT) (Lowe, 2004), and (2) machine learning classifiers, including support vector machine (SVM) (Ahmed et al., 2012) and k-nearest neighbor (KNN) (Li et al., 2009) classifiers. The goodness of the hand-designed feature components will affect the accuracy of the model. If incomplete or incorrect features are extracted from pest images, subsequent classifiers may not be able to distinguish between similar pest species.

With the continuous development of science and technology, deep learning technology has become a research hot spot of artificial intelligence. Image recognition technology based on deep learning improves the efficiency and accuracy of recognition, shortens the recognition time, reduces the workload of staff greatly, and lowers the cost. At present, pest identification methods based on deep learning technology are becoming more and more mature, and the scope of the research includes crops, plants, and fruits (Li and Yang, 2020; Liu and Wang, 2020; Zhu J. et al., 2021). However, the detection of forest pests faces many difficulties due to the lack of effective datasets. Some datasets are too small to meet the detection needs. Furthermore, most pest datasets are collected through traps or controlled laboratory environments, but they lack consideration of the real environment (Sun et al., 2018; Hong et al., 2021). Different species of pests may have a similar appearance. The same species may have different morphologies (nymphs, larvae, and adults) at different times (Wah et al., 2011; Krause et al., 2013; Maji et al., 2013).

For solving the problems mentioned above, we proposed a new forestry pest dataset for the forestry pest identification task. We collected pest data by searching through Google search engine and major forestry control websites. After filtering, we collected 2,278 original pest images covering adults, larvae, nymphs, and eggs of various pests. To alleviate the problem of category imbalance and improve the performance of the dataset for generalization ability, we took data enhancement operations, After data enhancement operations, the total amount of data increased to 7,163. For our pest dataset, we invited three experts in the field to assist us in classifying pests with the help of authoritative websites. Under the premise of knowing the category, we use the LabelImg annotation tool to annotate the image.

**Figure 1:**
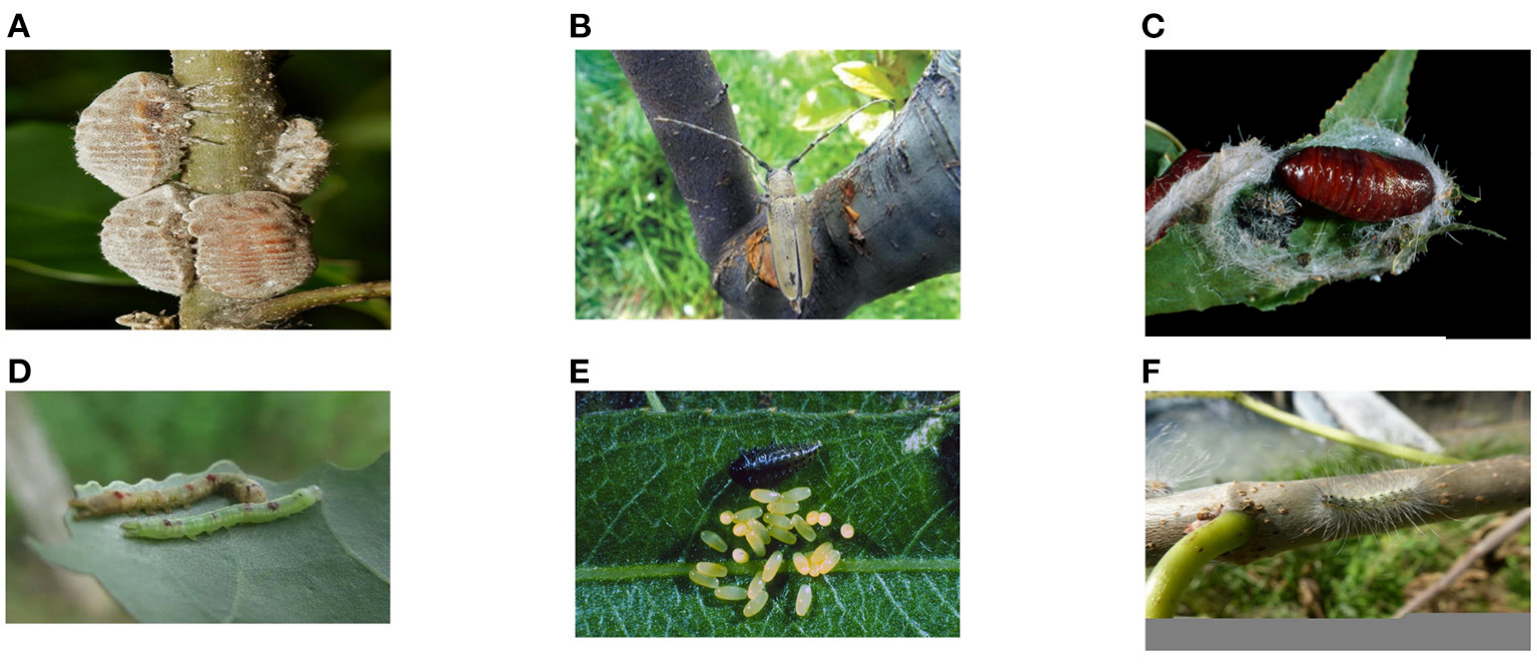
Sample images of Forestry Pest Dataset. **(A)** Drosicha contrahens; **(B)** Apriona germari; **(C)** Hyphantria cunea; **(D)** Micromelalopha troglodyta(Graeser); **(E)** Plagiodera versicolora(Laicharting); and **(F)** Hyphantria cunea larvae.

Our dataset covers 31 common forestry pests. We collected the forms of pests in different periods in the real wild environment. It meets the basic requirements of forestry pest identification. Figure 1 shows some examples of the dataset. To explore the application value of our proposed dataset, we use popular object detection algorithms to test the dataset.

The contributions of this work are summarized as follows:

1) We construct a new forestry pest dataset for the target detection task.
2) We tested our dataset on several popular object detection models. The results indicate that the dataset is challenging and creates new research opportunities. We hope this work will help advance future research on related fundamental issues as well as forestry pests identification tasks.

## 2. Related Works

In this section, we introduce the related work of agricultural pest identification and review the existing data sets.

### Pest Identification of Agriculture

Pest identification helps researchers improve the quality and yield of agricultural products. Earlier pest identification models are mainly based on machine learning techniques. For example, Le-Qing and Zhen (2012) utilizes local average color features and SVM to diagnose 10 insect pests based on a dataset containing 579 samples. Fina et al. (2013) combined K-mean clustering algorithm with adaptive filter for crop pest identification. Zhang et al. (2013) designed a field pest identification system and their dataset comprises approximately 270 training samples. Ebrahimi et al. (2017) used a differential kernel function SVM method for classification and detection, but the evaluated dataset is small, containing just 100 samples. Wang et al. (2018) uses digital morphological features and K-means to segment pest images.The above traditional pest identification algorithms have been studied with good results, but all of them have limitations, and their detection performance depends on the performance of the pre-designed manual feature extractor and the selected classifier.

Convolutional neural network (CNN) has strong image feature learning capability, such as ResNet (He et al., 2016) and GoogleNet (Szegedy et al., 2015) can learn deep higher-order features from images and can automatically learn shape, color, and texture of complex images and other multi-level features, overcoming the traditional manually designed feature extractors' limitations and subjectivity. It has obvious advantages in target detection, segmentation, classification of complex images, *etc*.

Liu and Wang (2020) constructed a tomato diseases and pests dataset and improved the YOLOV3 algorithm to detect tomato pests. Wang et al. (2020) introduced an attention mechanism in residual networks for improving the recognition accuracy of small targets. A two-stage aphid detector named Coarse-to-Fine Network (CFN) is proposed by Li et al. (2019) to detect aphids with different distributions. Zhu J. et al. (2021) uses super-resolution image enhancement technology and an improved YOLOv3 algorithm to detect black rot on grape leaves.

In general, CNN-based pest identification work can well avoid the limitations of traditional methods and improve the performance of pest identification. However, most target detection models have applied many hand-crafted components.To some extent, the parameters of the manual components increase the workload. To eliminate the impact of manual components on the model, researchers have considered using the versatile and powerful relational modeling capabilities of the transformer to replace the hand-crafted components. Carion et al. (2020) put forward the end-to-end object detection with transformers (DETR) by combining the convolutional neural network and the transformer, which built the first complete end-to-end target detection model and achieved highly competitive performance.

### Related Datasets

At present, deep learning-based agricultural pest identification and classification is maturing. The research scope includes a variety of cash crops such as crops, vegetables, and fruits, and relevant datasets have also been constructed.

Wu et al. (2019) constructed the IP102 pest dataset, which covers more than 70,000 images of 102 common crop pests. Wang et al. (2021) constructed the Agripest field pest dataset, which includes more than 49,700 images of pests in 14 categories. Hong et al. (2020) constructed a moth dataset by pheromone traps, which were labeled with four classes, including three moth classes and an unknown class of non-target insects. As a result of data collection and labeling, a total of 1,142 images were obtained. Liu Z. et al. (2016) constructed a rice pest dataset. The data were collected from image search databases of Google, Naver, and FreshEye, including 12 typical species of paddy field pest insects with a total of over 5,000 images. He et al. (2019) designed an oilseed rape pest image database, including a total of 3,022 images with 12 typical oilseed rape pests. Lim et al. (2018) build an insects dataset by specimens and Internet. The dataset consists of about 29,000 image files for 30 classes. Baidu constructed a forestry pest dataset that includes over 2,000 images for 7 classes through the specimen and traps. Chen et al. (2019) build a garden pests datasets. The dataset consists of about 9,070 image files for 38 classes. Liu et al. (2022) constructed a representative dataset of forest pests classification, including 67 categories and 67,953 original images. However, so far, only the dataset of Liu et al. (2022) is available for the detection of forest pests.

In conclusion, the research on crop diseases and insect pests based on deep learning covers a wide range, but in forestry, the detection and control of forest diseases and insect pests is still a challenge.

## 3. Our Forestry Pest Dataset

### Data Collection and Annotation

We collect and annotate the dataset with following four stages: 1) taxonomic system establishment, 2) image collection, 3) preliminary data filtering, 4) Data Augmentation, and 5) professional data annotation.

#### Taxonomic System Establishment

**Figure 2:**
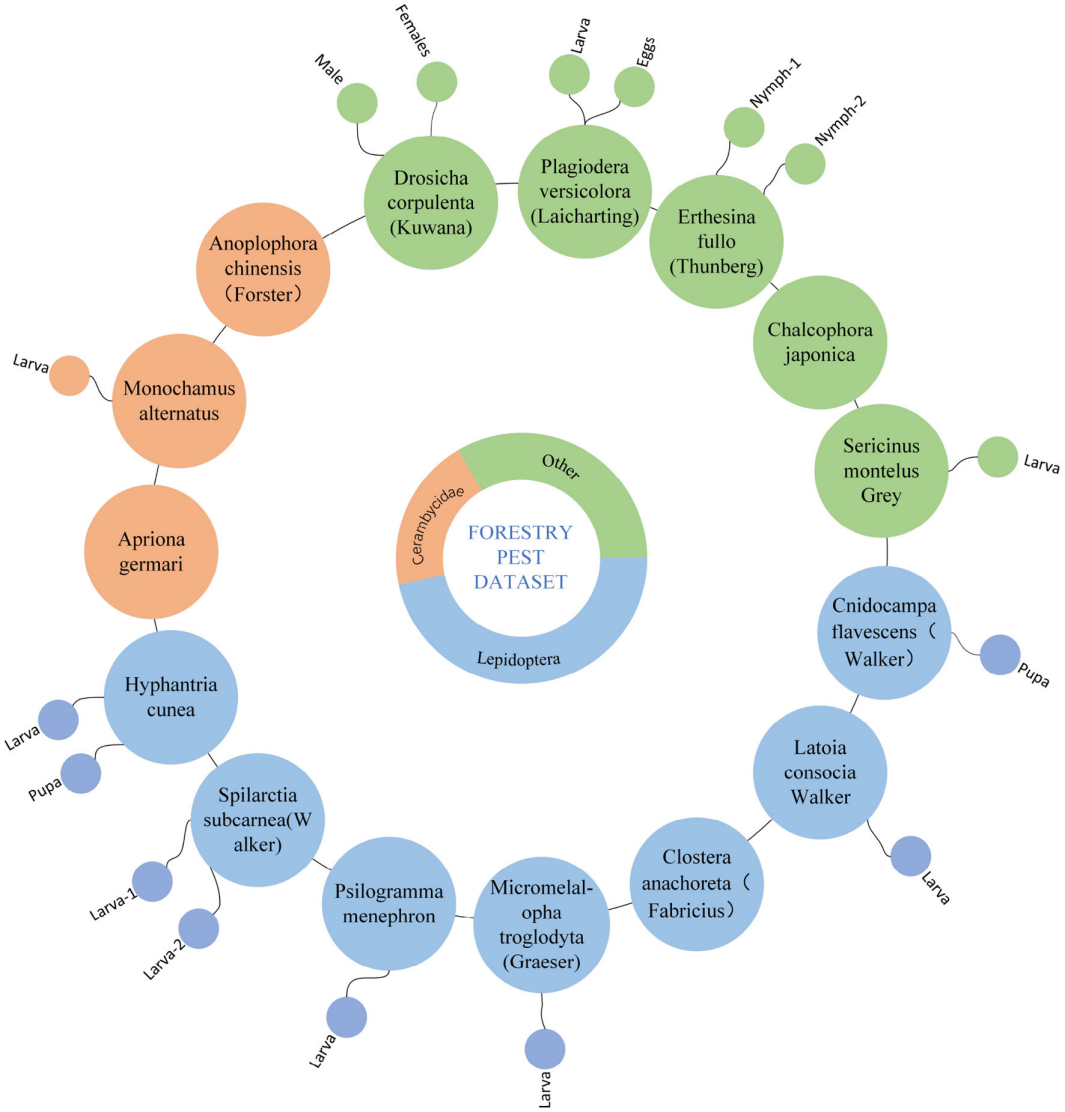
The classification structure of Forestry Pest Dataset.

We have established hierarchical classification criteria for the Forestry Pest Dataset. We asked three forestry experts to help us discuss common forest pest species. In addition, to better meet the needs of forest pest control, we use the larvae, eggs, and nymphs of each pest as subclasses, specifically, *Sericinus montela* and *Sericinus montela(larvae)* according to our The standards are divided into two categories. There are 31 classes finally obtained and they present a hierarchical structure as shown in Figure 2.

#### Image Collection

We utilize the Internet and forestry pest databases as the main sources of dataset images. We use the Chinese and scientific names of pests to search and save on common image search engines and also search for their corresponding eggs, larvae, and other images. Afterward, we searched for corresponding images from specialized agricultural and forestry pest websites.

#### Preliminary Data Filtering

From candidate images obtained from various websites and databases, we organized four volunteers to manually screen images. With the assistance of forestry experts, volunteers removed invalid and duplicate images that did not contain pests and repaired damaged images. And establish the initial category information. Specifically, in the initial pest collection work, we collected according to 15 categories, the purpose of this is to enhance the balance of data in the next step. Finally, we obtained 2,278 original images.

#### Data Augmentation

**Figure 3:**
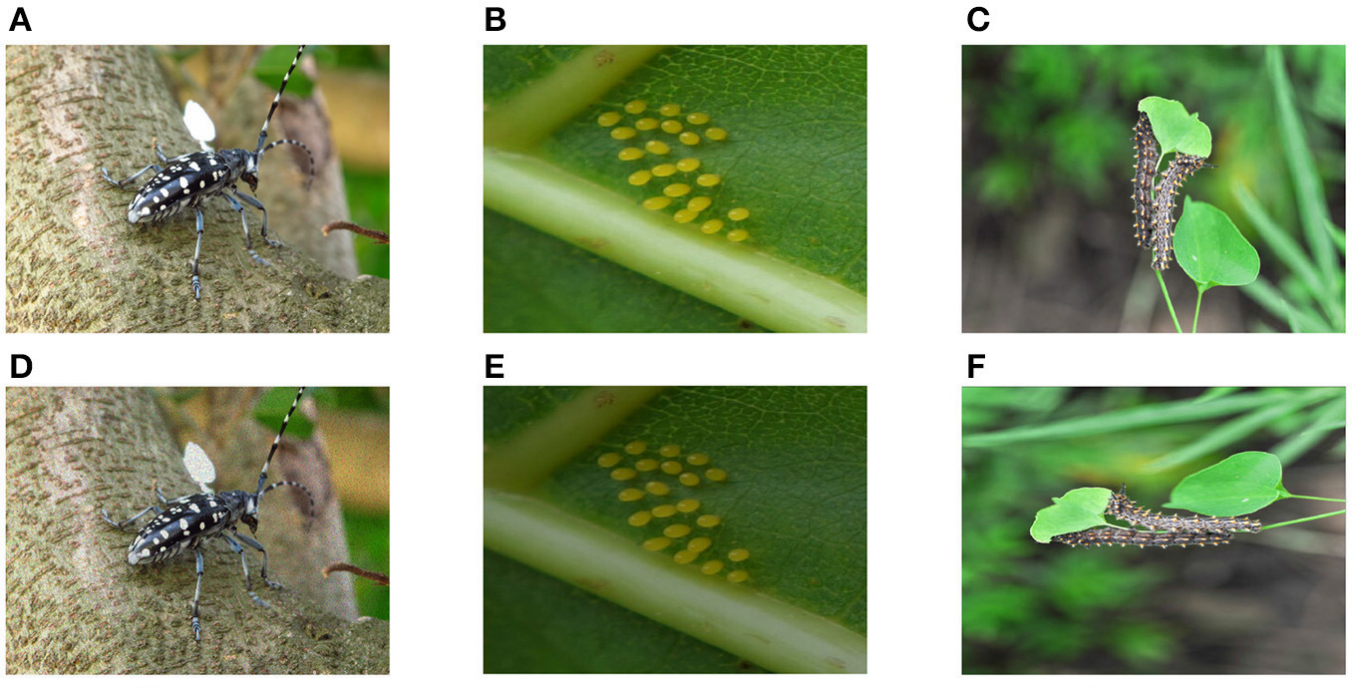
Example of image data enhancement method. The first row is the original image, and the second row corresponds to the enhanced image. **(A)** Original image, **(B)** Original image, **(C)** Original image, **(D)** Noise, **(E)** Brightness transformation, and **(F)** Rotation.

To ensure the effectiveness of the model and improve the generalization ability of the dataset, we use 7 image enhancement techniques such as rotation, noise, and brightness transformation to expand our dataset. For the species with less data, we adopt 7 methods for augmentation, and our purpose is to balance the number of pest images for each category. Figure 3 shows some examples of data augmentation. At the same time, we extract subclasses such as eggs, larvae, and nymphs under each category to establish subclass information. Finally, we obtained a forestry pest dataset of 31 categories (including 16 sub-categories) with a total of 7,163 images. Table 1 shows specific data for each category.

**Table 1:** Details of Forestry Pest Dataset.

**Class index**	**Pest**	**Sample size**
0	*Drosicha contrahens (female)*	218
1	*Drosicha contrahens (male)*	210
2	*Chalcophora japonica*	158
3	*Anoplophora chinensis*	426
4	*Psacothea hilaris(Pascoe)*	218
5	*Apriona germari(Hope)*	342
6	*Monochamus alternatus*	184
7	*Plagiodera versicolora(Laicharting)*	306
8	*Latoia consocia(Walker)*	290
9	*Hyphantria cunea*	303
10	*Cnidocampa flavescens(Walker)*	290
11	*Cnidocampa flavescens(Walker) (pupa)*	176
12	*Erthesina fullo*	280
13	*Erthesina fullo (nymph)*	156
14	*Erthesina fullo (nymph 2)*	192
15	*Spilarctia subcarnea(Walker)*	188
16	*Psilogramma menephron*	218
17	*Sericinus montela*	364
18	*Sericinus montela (larvae)*	200
19	*Clostera anachoreta*	294
20	*Micromelalopha troglodyta(Graeser)*	238
21	*Latoia consocia(Walker) (larvae)*	204
22	*Plagiodera versicolora(Laicharting) (larvae)*	196
23	*Plagiodera versicolora(Laicharting) (ovum)*	134
24	*Spilarctia subcarnea(Walker) (larvae)*	186
25	*Spilarctia subcarnea(Walker) (larvae 2)*	164
26	*Psilogramma menephron (larvae)*	208
27	*Cerambycidae (larvae)*	196
28	*Micromelalopha troglodyta(Graeser) (larvae)*	226
29	*Hyphantria cunea (larvae)*	224
30	*Hyphantria cunea (pupa)*	174

#### Professional Data Annotation

For object detection tasks, annotation information is very important, which is related to the recognition accuracy of the model. The first is to classify the collected pests. In the image collection stage, we already have the initial classification information. On this basis, our three experts first need to independently determine whether the image conforms to the category. Uncertain images are eliminated by three experts. The location information of pests is also very important, which can help forestry protection workers better find the specific location of pests. On the premise of understanding the types of pests, we use the LabelImg tool to label the images, mainly labeling the types and locations of pests.

We recruited three volunteers to assist us in the annotation of the data. First, each volunteer will receive guidance and training from three forestry professionals to understand the basic characteristics of each type of pest. After that, we will train the three volunteers to use the LabelImg tool. Volunteers need to master the basic usage of LabelImg, including importing files and adding, modifying, and deleting annotation information. Experts will assist volunteers to annotate some images in the early stage, and then volunteers will independently complete subsequent image annotations. In the process of annotation, images that are difficult to identify or annotate will be resolved through consultation by three experts. After all image annotations are completed, volunteers use the annotation visualization to check whether there is any wrong or defective annotation information and submit it to experts for the final ruling.

### Dataset Split

Our Forestry Pest Dataset contains 7,163 images and 31 pest species. To ensure the training results, we randomly divide according to the following ratio: (Train: Val=9: 1): Test=9: 1. Specifically, the Forestry Pest Dataset is split into 5,801 training, 645 validation, and 717 testing images for the object detection task.

### Comparison With Other Forestry Pest Datasets

In Table 2, we compare our dataset with some existing datasets related to forestry pest identification tasks. Sun et al. (2018) and Hong et al. (2021) created related datasets using pheromone trap collection, but their datasets only deal with specific species of pests. The forestry pest dataset proposed by Baidu is processed and collected in a controlled laboratory environment. Due to these limitations, these related datasets are difficult to apply to practical applications. Chen et al. (2019) and Liu et al. (2022) focus on the classification of forest pests. Their dataset is rich in pest species and has a sufficient number of samples, which has played a huge role in practical applications. However, they have not made relevant attempts on pest detection tasks, and the relevant datasets have not been published.

**Table 2:** Comparison with existing forestry pest datasets.

**Dataset**	**Year**	**Class**	**Sample size**	**Avg**	**Public**
Sun et al. (2018)	2018	1	2,183	-	Y
BaiDu	2019	7	2,183	311	Y
Chen et al. (2019)	2019	38	9,072	238	N
Hong et al. (2021)	2021	1	50	-	N
Liu et al. (2022)	2022	67	67,953	1,014	N
Ours	2022	31	7,163	231	Y

*The “Class” denotes the number of categories. The “Public” indicates if the dataset is open source and available. The “Y” and “N” denote “yes” and “no,” respectively. The “Avg” denotes average numbers of samples per class*.

### Diversity and Difficulty

Pests with different life cycles have different degrees of damage to forestry, so we retained images of these different morphological pests during data collection and annotation. However, due to the small differences between classes (similar features) and large differences within classes (there are many stages in the life cycle) of pests, accurate classification of their features is a difficult task in detection tasks. In addition, the imbalanced data distribution brings challenges to the feature learning of the model, and the imbalanced data will cause the learning results of the model to be biased toward a relatively large number of classes.

## 4. Experiment

To explore the application value of our proposed dataset, we evaluate several popular object detection algorithms on this dataset. Based on the two-stage approach of Faster RCNN (Ren et al., 2015), they scan the feature maps for potential objects by sliding windows, then classify them and regress the corresponding coordinate information. YOLOV4 (Bochkovskiy et al., 2020) and SSD (Liu W. et al., 2016) based on one-stage methods directly regress category and location information. In addition, we also evaluate the transformer-based end-to-end object detection algorithm Deformable DETR (Zhu X. et al., 2021).

### Experimental Settings

The framework used for this experiment is python3.8, torch1.9, cuda11.1. The experimental hardware is shown in Table 3.

**Table 3:** Configuration of experimental environment.

**Hardware**	**Model**
CPU	i7–8,700
Memory	64GB
GPU	RTX 3,090 24GB
Hard disk	2.5TB

### Object Detection Algorithms

After the accumulation of R-CNN and Fast RCNN, Faster RCNN integrates feature extraction (feature extraction), proposal extraction, bounding box regression (rect refine), and classification into one network in structure, which greatly improves the comprehensive performance., especially in terms of detection speed. SSD is a single-stage target detection algorithm, which uses convolutional neural network for feature extraction, and takes different feature layers for detection output. SSD is a multi-scale detection method. Based on the original YOLO target detection architecture, the YOLOV4 algorithm adopts the best optimization strategy in the CNN field in recent years, and has different degrees of optimization in terms of data processing, backbone network, network training, activation function, loss function, etc., achieving the perfect balance of speed and precision. Based on DETR, Deformable DETR improves the calculation method of the attention mechanism through sparse sampling, reduces the amount of calculation, and greatly reduces the training time of the model while ensuring accuracy.

### Parameters of Model Training

SSD, Faster RCNN, YOLOV4, and Deformable DETR initial model parameter settings are shown in Tables 4, 5. To take into account the accuracy and training time, in the previous Deformable DETR model training process, the model reached convergence around 150 epoch, therefore, we chose 150 epoch, and Deformable DETR performed a learning rate decay every 40 epoch, so we chose 80 epoch as the intermediate result, Compare the performance of the four models on the dataset. At the same time, to maintain the consistency of the training cycle, we set the same epoch as Deformable DETR for the other three models.

**Table 4:** Model parameter settings of SSD, Faster RCNN, and YOLOV4.

**Name**	**Value**
Batch size	16
Epoch	150
Learn rate	0.0001
NMS	0.3
Match threshold	0.5

**Table 5:** Model parameter settings of Deformable DETR.

**Name**	**Value**
Batch size	2
Epoch	150
Learn rate	0.00002

### Evaluation Metrics

We use *mAP* and *Recall* as evalution metrics which are two widely used metrics in target detection. *mAP* and *Recall* are calculated as follows:


(1) $$\begin{array}{r} Precision=\frac{TP}{TP+FP} \end{array}$$

(2) $$\begin{array}{r} Recall=\frac{TP}{TP+FN} \end{array}$$

(3) $$\begin{array}{r} mAP_{\alpha }=\frac{1}{N}\overset{N}{\underset{n=1}{\sum }}AP_{\alpha }^{n} \end{array}$$

(4) $$\begin{array}{r} mAP_{multi-scale}=\frac{1}{N_{ms}}\frac{1}{10}\overset{N_{ms}}{\underset{n_{ms}}{\sum }}\overset{0.95}{\underset{\alpha =0.5,step=0.05}{\sum }}mA\overset{\alpha }{\underset{ms}{P}} \end{array}$$

Where, *TP* is a positive sample predicted by the model as a positive class, *FP* is a negative sample predicted as a positive class by the model, *FN* is a negative class predicted by the model positive sample. Each class can calculate its Precision and Recall, and each class can get a PR curve, and the area under the curve is *AP*. *mAP*_α_ and *mAP*_*multi*−*scale*_ are the average of all classes *AP* at different confidence levels α and different scales value.

In the MS COCO dataset, objects with an area less than 32*32 are considered small objects, while objects with an area greater than 32*32 and less than 96*96 are considered medium objects.

### Experimental Results

Average precision performance of object detection methods under different IoU thresholds. The results are shown in Table 6.

**Table 6:** mAP_α_ values of different models on Forestry Pest Dataset.

**Model**	**Epoch**	***mAP*_0.5_**	***mAP*_0.75_**
SSD	80	96.6	80.6
Faster RCNN	80	96.8	83.6
YOLOV4	80	98.8	70.2
Deformable DETR	80	96.6	89.8
SSD	150	98.1	91.1
Faster RCNN	150	97.5	85.2
YOLOV4	150	99.7	88.3
Deformable DETR	150	97.1	90.4

**Figure 4:**
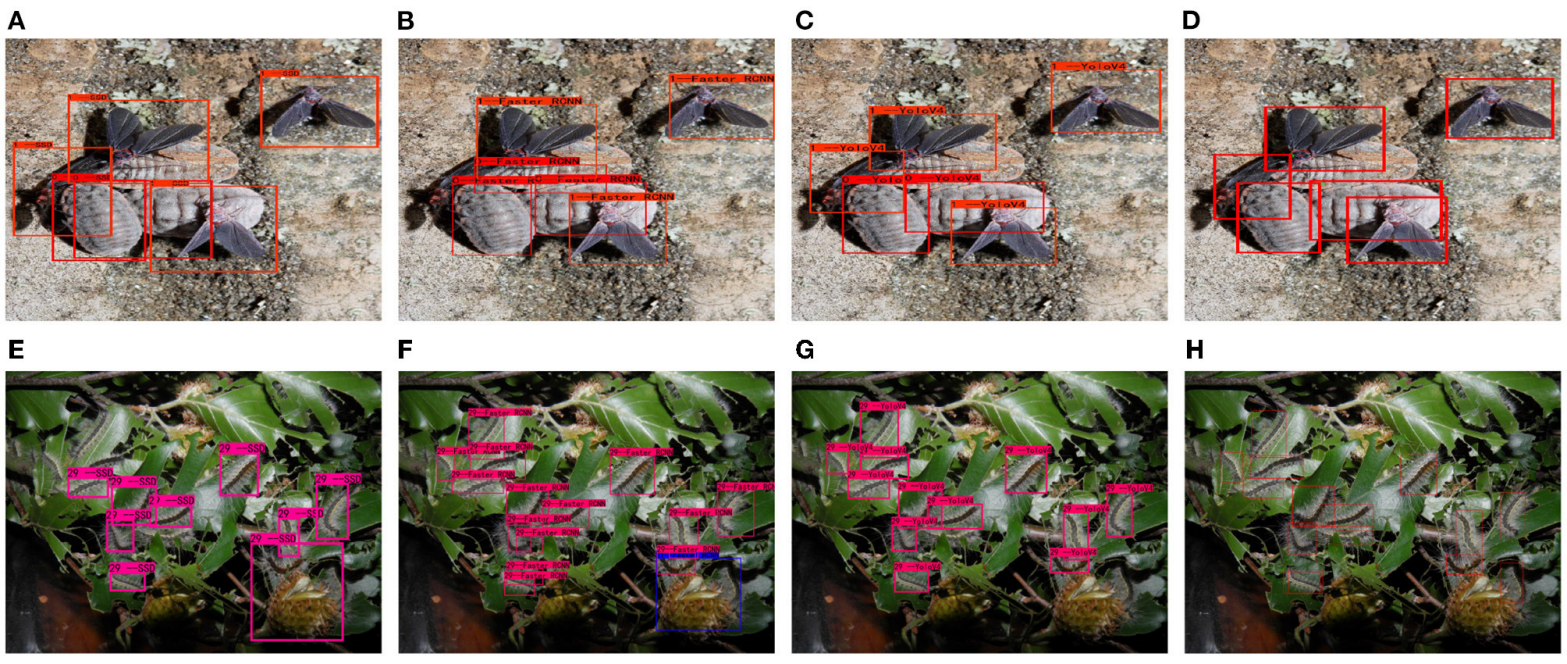
Sample detection results on adults and larvae. From left to right are **(A,E)** SSD, **(B,F)** Faster RCNN, **(C,G)** YOLOV4, and **(D,H)** Deformable DETR.

From the experimental results in Table 6, it can be seen that the dataset in this paper has good accuracy on mainstream target detection models under short-time training. The recently proposed Deformable DETR can also be used on the dataset in this paper. Achieve roughly the same performance as SSD, Faster RCNN, and YOLOV4. An example of the detection of the model is shown in Figure 4.

From the above results, Deformable DETR based on Transformer architecture does not perform as well as YOLOV4 or even Faster RCNN in some cases. Based on our analysis, there are the following reasons.

1) Deformable DETR has no prior information. Whether it is YOLOV4 or Faster RCNN, they all have a part of prior information input, such as the clustering results of the coordinate information of the dataset, which can help the model find the target faster.
2) Although the attention mechanism calculation of Deformable DETR has been improved, its essence is still based on pixel calculation, which leads to a huge amount of calculation for high-resolution images. Deformable DETR does not have a feature fusion module similar to YOLOV4, which is detrimental to the detection of small objects.
3) Deformable DETR uses the Hungarian matching algorithm to match the prediction and ground truth, which cannot guarantee the convergence and accuracy of the model to a certain extent.

### Confusion Matrix

**Figure 5:**
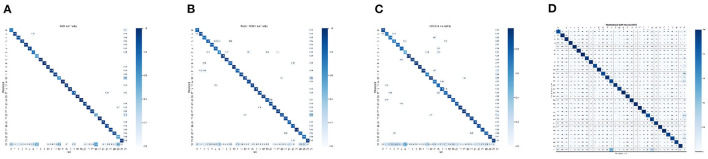
Confusion matrix of the mo del on the test set. Epoch=150, Iou-threshold=0.5, Index=31 means background. **(A)** SSD, **(B)** Faster RCNN, **(C)** YOLOV4, and **(D)** Deformable DETR.

The confusion matrix in target detection is very similar to that in classification, but the difference is that the object of the classification task is a picture, while the detection task is different. It includes two tasks of positioning and classification, and the object is each target in the picture. Therefore, to be able to draw positive and negative examples in the confusion matrix, it is necessary to distinguish which results are correct and which are wrong in the detection results. At the same time, the detection of errors also needs to be classified into different error categories. How to judge whether a detection result is correct, the most common way at present is to calculate the IOU of the detection frame and the real frame, and then judge whether the two frames match according to the IOU. For some targets below the threshold or not detected, they will be considered as the background class. The confusion matrix results of the model on the test set are shown in Figure 5.

### Case Study: Experiment on Large, Medium, and Small Targets

**Figure 6:**
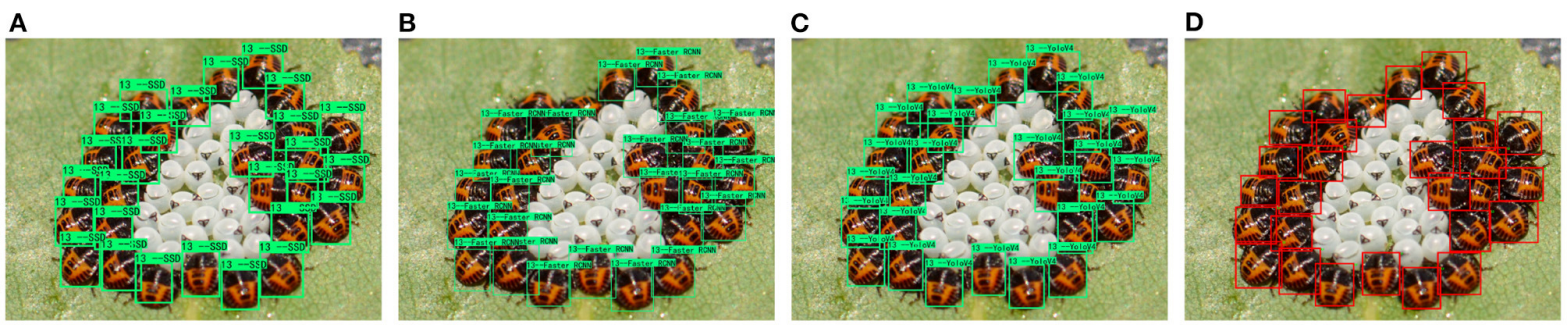
Small sample detection results on our Forestry Pest Dataset. **(A)** SSD, **(B)** Faster RCNN, **(C)** YOLOV4, and **(D)** Deformable DETR.

Small targets have always been a difficult task in target detection due to their small size and lack of feature information. In the field of forest pest detection, the detection of small targets is also a difficult task due to the real complexity. Our dataset contains small objects such as larvae and eggs. We also consider the model's ability to detect small objects in our dataset. The results are shown in Tables 7, 8. The detection example of each model on small targets is shown in Figure 6.

**Table 7:** mAP_multi−scale_ values of multi-scale results achieved by different models on Forestry Pest Dataset.

**Model**	**Epoch**	**mAP_*small*_**	***mAP*_*medium*_**	***mAP*_*large*_**
SSD	80	27.4	53.5	72.7
Faster RCNN	80	14.2	49.0	74.0
YOLOV4	80	49.4	57.0	62.2
Deformable DETR	80	28.0	61.6	87.1
SSD	150	35.2	65.4	84.7
Faster RCNN	150	30.0	48.9	76.5
YOLOV4	150	56.2	63.1	73.2
Deformable DETR	150	30.3	63.8	87.7

**Table 8:** Recall_multi−scale_ values of multi-scale results achieved by different models on Forestry Pest Dataset.

**Model**	**Epoch**	***Recall*_*small*_**	***Recall*_*medium*_**	***Recall*_*large*_**
SSD	80	41.2	61.5	77.1
Faster RCNN	80	23.8	55.8	78.1
YOLOV4	80	53.0	61.0	67.7
Deformable DETR	80	31.4	68.8	91.3
SSD	150	44.9	69.6	87.4
Faster RCNN	150	38.8	54.7	80.0
YOLOV4	150	60.2	67.5	77.0
Deformable DETR	150	34.3	71.1	91.6

As can be seen from the above table, YOLOV4 significantly leads the rest of the models in the detection of small targets, thanks to its powerful network structure and feature fusion, Deformable DETR is based on the attention mechanism of pixel-level computing, and the detection of small targets is not very friendly.

## 5. Conclusion and Future Directions

### Conclusion

In this work, we collect a dataset, for forest insect pest recognition, including over 7,100 images of 31 classes. Compared with previous datasets, our dataset focuses on a variety of forestry pests, meets the detection needs of both real and experimental environments, and also includes pest forms in different periods, which some previous forestry pest datasets neglected. Meanwhile, we also evaluate some state-of-the-art recognition methods on our dataset. Exceptionally, this dataset has received good feedback on some mainstream object detection algorithms. However, in the detection of small objects, the existing deep learning methods cannot achieve the desired accuracy. Inspired by the success of the application in computer vision of the Transformer model, we also introduced the Transformer model to solve the forestry pest identification problem. We hope this work will help advance future research on related fundamental issues as well as forestry pests identification tasks.

### Future Directions

To better promote the development of forestry pest identification, we will continue to collect forestry pest data and expand the dataset to 99 categories. For pests that have occurred or diseases caused by pests, there is a lack of relevant data sets and research support. In response, we will collect images of diseases caused by insect pests.

Although the existing deep learning models have achieved good results in forest pest identification, small target recognition is still a challenge. We will optimize and improve the model in the follow-up to further improve the model's ability to detect small targets.

## Data Availability Statement

The datasets for this study can be found in the https://drive.google.com/drive/folders/1WnNDLEZCNpXKwJzjnJsQKSAYKljIIRCH?usp=sharing.

## Author Contributions

GW designed research and revised the manuscript. LL and BL conducted experiments, data analysis, and wrote the manuscript. RZ collected pest data. WC and RD revised the paper. All authors contributed to the article and approved the submitted version.

## Funding

The research is supported by the Educational Department of Jilin Province of China (Grant No. JJKH20210752KJ). The research is also supported by the project of research on independent experimental teaching mode of program design foundation based on competition to promote learning which is the Higher Education Research of Jilin Province of China (Grant No. JGJX2021D191).

## Conflict of Interest

The authors declare that the research was conducted in the absence of any commercial or financial relationships that could be construed as a potential conflict of interest.

## Publisher's Note

All claims expressed in this article are solely those of the authors and do not necessarily represent those of their affiliated organizations, or those of the publisher, the editors and the reviewers. Any product that may be evaluated in this article, or claim that may be made by its manufacturer, is not guaranteed or endorsed by the publisher.
